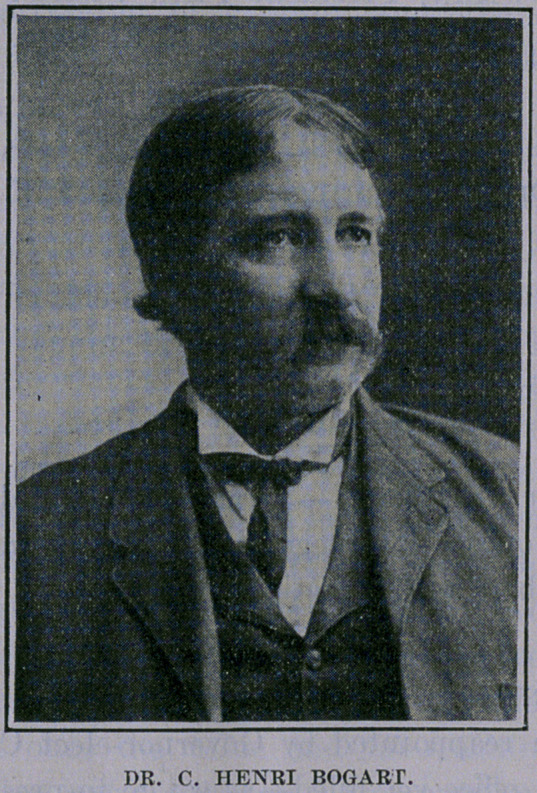# Editorialets

**Published:** 1910-12

**Authors:** 


### Editorialets.

APPRECIATION.—Move me up two years. I enjoy the "Red Back." W. G. Jameson, Palestine.

---

I would not be without it for five times what it costs. The last issue is worth many times the cost of the JOURNAL. Wishing you much success in your efforts to advance the cause of organized medicine, I am, fraternally, L. H. Reeves, Decatur.

---

Let the "Red Back" come on, and may you be spared many years to fight the battle that you have so gallantly waged for freedom in medicine. W. P. White, Henderson.

---

DR. J. H. EASTLAND, of Mineral Wells, Texas, is at the New York Polyclinic, New York.

---

DR. A. R. BOWMAN, President Fifth District Medical Society (Bexar county district), has removed to El Paso.

---

DIED.—Dr. James T. Black, of Elroy, Texas, died November 9th (ult.), at Dripping Springs, Texas, aged 35.

---

DR. A. L. LINCECUM, of El Campo, was elected President of the Eighth District Medical Society at the annual meeting held at Bay City, October 27th.

---

DRS. BENNETT AND SCOTT—in an auto—collided with an auto driven by Dr. Jones, of Pfluegerville. Dr. Bennett had two ribs broken. The others were practically unhurt.

---

DR. JOHN PRESTON, Superintendent State Lunatic Asylum at Austin, has been reappointed by Governor-elect Colquitt. This is the only medical office yet filled—as we go to press.

---

MARRIED at Wharton, Texas, the home of the bride, December 1st (inst.), Miss Lurline Andrews, daughter of Dr. and Mrs. J. M. Andrews, to Mr. Albert Hodges Wadsworth, of Matagorda, Texas. Cards received.

---

DR. FLY'S EXCELLENT PAPER in our last issue was read by invitation at the meeting of the San Angelo District Medical Society and not the San Antonio, as stated. Fault of the printer and the proof reader.

---

DR. ISADORE DYER, of New Orleans, was elected President of the Southern Medical Association at the recent Nashville meeting, and Dr. E. V. Depew, of San Antonio, was elected President of the Fifth Councilor's District Medical Society at the recent annual meeting at San Antonio.

---

WANTED.—A graduated and licensed physician, especially experienced in physical therapeutics, including electric and hydrotherapy, and expert in the use of the appliances for such treatment, wishes a position as such in a sanatorium, or with a physician needing such assistant. Address Dr. H. C., care this JOURNAL.

---

DR. C. HENRI BOGART.

DR. C. HENRI BOGART, Brookville, Ind. Born at Cincinnati, October 26, 1857. Physician, lecturer, reformer, poet. Dr. Bogart's writings published in the TEXAS MEDICAL JOURNAL and elsewhere have attracted wide attention, he being a pioneer in the reform movement to save the integrity of the race by preventing the propagation of the unfit. Read his splendid article in this issue. He was the author of the Indiana Sterilization Law, now so famous, and which law other States have adopted, and Texas will try for next Legislature.

---

PHYSIOLOGIC THERAPEUTICS, the live, new journal published by Dr. Henry R. Harrower, of Chicago, will celebrate the New Year with a special double number. We learn that several thousand extra copies will be printed and sent with the season's greetings to such physicians as may be interested in seeing this able exponent of the progress in the non-medicinal methods of treatment. From the advanced program which we have received it would seem that this number will be an especially fine one. Those of our readers who desire a copy should send a postal request to Dr. H. R. Harrower, Park Ridge, Illinois.

---

Dr. John Veitch Shoemaker, of Philadelphia, died on Tuesday, October 11th, aged 58 years. His health had been failing for a number of months, and about a year ago he had an attack of apoplexy. After the stroke he was soon able to attend to professional work again, and he resolutely performed it almost to the last. He was a graduate of the Jefferson Medical College, of the class of 1874. He soon became a lecturer in the Philadelphia School of Anatomy and subsequently in the Jefferson Medical College. A number of years later he was active in founding the Medico-Chirurgical College, in which he remained a professor up to the time of his death. He was a dermatologist of distinction, but still more a general therapeutist. For many years he was the editor of the *Medical Bulletin*, and he frequently wrote for other medical journals. For considerable period he served as surgeon general of the National Guard of the State of Pennsylvania.

---

A Swindler Abroad.—Hotels, druggists, physicians, liverymen and others are warned against a man traveling from place to place presenting a card with the name "R. F. Hall" printed in the center. In the lower left-hand corner are the words "Parke, Davis & Co., and in the lower right-hand corner the words "Detroit, Mich." This man is described as follows: "5 feet 6 or 8 inches, 150 pounds, fiend for Turkish cigarettes, about 27 years, complexion medium, wears nose glasses and continually takes them off and on; he is a swell dresser, good talker, fine appearance, wears one of those light-colored slip on or off raincoats." This individual has no connection with Parke, Davis & Co., and so far as heard uses the card to facilitate the passing of bogus checks. Because of incidents like these nearly all concerns employing "drummers" forbid them to borrow money or seek credit, except upon individual responsibility and acquaintanceship. Therefore, those seeking credit or loans, especially from comparative strangers, on the strength of their alleged connection with some important concern, should be treated as imposters.

## Figures and Tables

**Figure f1:**